# CHCHD2 rescues the mitochondrial dysfunction in iPSC-derived neurons from patient with Mohr-Tranebjaerg syndrome

**DOI:** 10.1038/s41419-025-07472-9

**Published:** 2025-03-12

**Authors:** Yihua Huang, Zirui Chen, Weiling Deng, Yawei Jiang, Yue Pan, Zhirong Yuan, Hailiang Hu, Yongming Wu, Yafang Hu

**Affiliations:** 1 Department of Neurology, Nanfang Hospital, Southern Medical University, Guangzhou, China; 2 Institute of Brain Disease, Nanfang Hospital, Southern Medical University, Guangzhou, China; 3 Department of Biochemistry, School of Medicine, Southern University of Science and Technology, Shenzhen, China

**Keywords:** Neurodegeneration, Neurodegeneration

## Abstract

Mohr-Tranebjaerg syndrome (MTS) is a rare X-linked recessive neurodegenerative disorder caused by mutations in the *Translocase of Inner Mitochondrial Membrane 8A (TIMM8A)* gene, which encodes TIMM8a, a protein localized to the mitochondrial intermembrane space (IMS). The pathophysiology of MTS remains poorly understood. To investigate the molecular mechanisms underlying MTS, we established induced pluripotent stem cells (iPSCs) from a male MTS patient carrying a novel *TIMM8A* mutation (c.225-229del, p.Q75fs95*), referred to as MTS-iPSCs. To generate an isogenic control, we introduced the same mutation into healthy control iPSCs (CTRL-iPSCs) using the Clustered Regularly Interspaced Short Palindromic Repeats/CRISPR-associated protein 9 (CRISPR/Cas9), resulting in mutant iPSCs (MUT-iPSCs). We differentiated the three iPSC lines into neurons and evaluated their mitochondrial function and neuronal development. Both MTS- and MUT-iPSCs exhibited impaired neuronal differentiation, characterized by smaller somata, fewer branches, and shorter neurites in iPSC-derived neurons. Additionally, these neurons showed increased susceptibility to apoptosis under stress conditions, as indicated by elevated levels of cytochrome c and cleaved caspase-3. Mitochondrial function analysis revealed reduced protein levels and activity of complex IV, diminished ATP synthesis, and increased reactive oxygen species (ROS) generation in MTS- and MUT-neurons. Furthermore, transmission electron microscopy revealed mitochondrial fragmentation in MTS-neurons. RNA sequencing identified differentially expressed genes (DEGs) involved in axonogenesis, synaptic activity, and apoptosis-related pathways. Among these DEGs, *coiled-coil-helix-coiled-coil-helix domain-containing 2* (*CHCHD2*), which encodes a mitochondrial IMS protein essential for mitochondrial homeostasis, was significantly downregulated in MTS-neurons. Western blot analysis confirmed decreased CHCHD2 protein levels in both MTS- and MUT-neurons. Overexpression of CHCHD2 rescued mitochondrial dysfunction and promoted neurite elongation in MTS-neurons, suggesting that CHCHD2 acts as a downstream effector of TIMM8a in the pathogenesis of MTS. In summary, loss-of-function of TIMM8a leads to a downstream reduction in CHCHD2 levels, collectively impairing neurogenesis by disrupting mitochondrial homeostasis.

**Figure:**
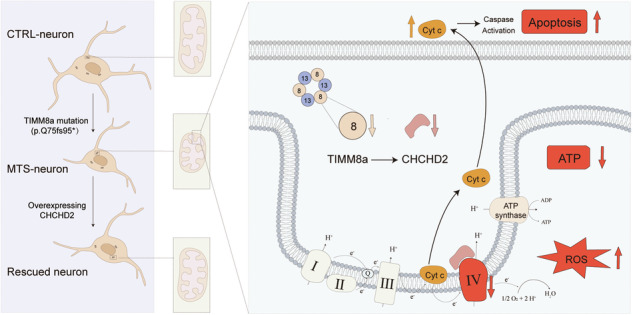
TIMM8a mutation (p.Q75fs95*) leads to mitochondrial dysfunction and neuronal defects in iPSC-derived neurons from patient with Mohr-Tranebjaerg syndrome, which are rescued by overexpression of CHCHD2. TIMM8a translocase of inner mitochondrial membrane 8a, CHCHD2 coiled-coil-helix-coiled-coil-helix domain-containing protein 2, MTS Mohr–Tranebjaerg syndrome, I mitochondrial complex I, II mitochondrial complex II, III mitochondrial complex III, IV mitochondrial complex IV, Q coenzyme Q10, Cyt c cytochrome c.

TIMM8a mutation (p.Q75fs95*) leads to mitochondrial dysfunction and neuronal defects in iPSC-derived neurons from patient with Mohr-Tranebjaerg syndrome, which are rescued by overexpression of CHCHD2. TIMM8a translocase of inner mitochondrial membrane 8a, CHCHD2 coiled-coil-helix-coiled-coil-helix domain-containing protein 2, MTS Mohr–Tranebjaerg syndrome, I mitochondrial complex I, II mitochondrial complex II, III mitochondrial complex III, IV mitochondrial complex IV, Q coenzyme Q10, Cyt c cytochrome c.

## Introduction

Mohr-Tranebjaerg syndrome (MTS, MIM: #304700), caused by mutations in *Translocase of Inner Mitochondrial Membrane 8* *A*/*Deafness Dystonia Protein 1* (*TIMM8A/DDP1*), is a rare X-linked recessive disorder characterized by progressive sensorineural hearing impairment starting in early childhood, gradually followed by a broader phenotypic spectrum, including ataxia, dystonia and visual disability [1–4]. Postmortem analysis of brain samples from a limited number of patients indicates neurodegeneration, including focal loss of neurons in the striatum with astrocytosis [5], moderate cortical atrophy in the frontal cortex [6], neuronal cell death in the visual cortex [7], and nearly complete loss of cochlear neuronal cells along with severe loss of vestibular neurons [8]. At present, there is still no effective therapy for MTS due to the poor understanding of its pathophysiology.

The human *TIMM8A* gene consists of one intron and two exons, encoding a 97-amino acid protein, TIMM8a/DDP1, which is located in the intermembrane space (IMS) of mitochondria [9]. Tim8p, the yeast homolog of TIMM8a, forms a hexamer with Tim13p to participate in cross-membrane transport. However, it remains controversial whether the same mechanism exists in humans [10–12]. Lymphoblasts from an MTS patient carrying the TIMM8a p.Q34X mutation showed reduced ATP production due to dysfunction of the Ca^2+^-binding aspartate/glutamate carrier, which is a substrate of the human Tim8a-Tim13 complex [13]. Other studies have found mitochondrial morphological alterations in fibroblasts derived from MTS patients with p.C66W and p.Met1Leu mutations [14, 15]. Human neuroblastoma cells (SH-SY5Y) with *TIMM8A* silencing, but not human embryonic kidney 293 cells (HEK293), exhibit defects in mitochondrial complex IV assembly, priming the cells for apoptosis [16, 17]. In a *Timm8a1* mutant mouse line, we demonstrated that mutant Tim8a leads to reduced weight gain, hearing impairment, anxiety-like behaviors, and cognitive defects, along with abnormal mitochondrial structure in the primary auditory cortex and hippocampus [18, 19]. Although these findings suggest that mutations in *TIMM8A* lead to mitochondrial dysfunction, the detailed mechanisms remain to be further elucidated.

In this study, we aimed to elucidate the molecular mechanisms underlying MTS by differentiating induced pluripotent stem cells (iPSCs) from an MTS patient into neurons. We found that the TIMM8a-CHCHD2 pathway is essential for maintaining mitochondrial homeostasis, and its downregulation led to impaired neuronal differentiation.

## Materials and methods

### Sanger sequencing of the mutation in *TIMM8A*

The forward and reverse primers for PCR amplification and Sanger sequencing of the mutation in *TIMM8A* (c.225-229del) are as follows:

- Forward primer: 5’-TGTAAGGCTAGAGAACATTGCT-3’;
- Reverse primer: 5’-CCTACTTCTGCCCTTTCTGATAA-3’.

PCR-amplified DNA fragments were analyzed by Sanger sequencing and compared with the *TIMM8A* genomic sequence (RefSeq NG_011734.1; https://www.ncbi.nlm.nih.gov/nuccore/NG_011734.1).

### Generation of iPSCs from peripheral blood mononuclear cells (PBMCs)

To generate iPSCs from a male MTS patient (MTS-iPSCs) and a male donor (CTRL-iPSCs), PBMCs were isolated from 8 mL blood samples using BD Vacutainer® CPT™ Cell Preparation Tube with Sodium Heparin (BD Biosciences, Cat# 362753). The PBMCs were then cultured in 24-well plates with complete PBMC medium at 37 °C in a humidified atmosphere with 5% CO_2_ for 4 days before transduction. On Day 0, the cells were transduced with the CytoTune™-iPS 2.0 Sendai Reprogramming Kit (Thermo Fisher Scientific, Cat# A16517). On Day 2, the cells were spun down to remove the virus-containing supernatant, resuspended in PBMC medium, and transferred to a low-attachment 24-well plate for further culture. On Day 4, the cells were plated onto Matrigel-coated culture dishes (Corning, Cat# 356234) and maintained in mTeSR™ Plus medium (STEMCELL Technologies, Cat# 100-0276). From Day 4 to Day 26, the medium was fully replaced every other day, and the plates were monitored under a microscope for the emergence of cell clumps indicative of reprogrammed cells. On Day 26, the iPSCs colonies were harvested and transferred to Matrigel-coated 24-well plates, with fresh medium replaced daily. Once the iPSCs colonies reached 80% confluence, they were dissociated using Accutase (STEMCELL Technologies, Cat# 07920) and passaged at a 1:8 ratio.

### CRISPR/Cas9-mediated generation of the *TIMM8A* mutation in CTRL-iPSCs

To generate an isogenic control (MUT-iPSCs) carrying the same *TIMM8A* mutation as our MTS patient, we used the CRISPR/Cas9 to edit the *TIMM8A* gene in CTRL-iPSCs. A pX330-2A-Puro plasmid (Guangzhou Institutes of Biomedicine and Health, GIBH, Guangzhou, China), containing the puromycin resistance gene cassette, was used to express Cas9 protein and the single guide RNA (sgRNA). The sgRNA sequences targeting *TIMM8A* were designed on the CRISPOR website (http://crispor.tefor.net/) as follows:

- Forward: 5’-CACCGCCAGTCGATTCAAGATGAAC -3’;
- Reverse: 5’-AAACGTTCATCTTGAATCGACTGGC-3’.

A single-stranded oligodeoxynucleotides (ssODN) [5’-ATGGACAAGCCTGGGCCAAAGTTGGACAGTCGGGCTGAGGCCTGTTTTGTGAACTGCGTTGAGCGCTTCATTGATACAAGCCATCTTGAATCGACTGGAACAGACCCAGAAATCCAAGC-3’] was designed as a template to introduce the *TIMM8A* mutation (c.225-229del). For gene editing, 1 × 10^6^ iPSCs were electroporated with 500 pmol of ssODN and 4 μg of the pX330 plasmid containing the sgRNA using Human Stem Cell Nucleofector Kit (Lonza, Cat# VPH-5022). The electroporated iPSCs were then plated onto Matrigel-coated six-well plates in mTeSR™ Plus medium supplemented with 10 μM Y-27632 (Selleck Chemicals, Cat# S1049). Positive clones were selected by mTeSR™ Plus medium supplemented with 1 μg/ml puromycin (Beyotime, Cat# ST551). Surviving cells were expanded into clones, collected, and analyzed by Sanger sequencing.

### Teratoma formation

1 × 10^6^ iPSCs mixed with Matrigel were subcutaneously injected into NOD-SCID mice (Gempharmatech, Jiangsu, China) and allowed to differentiate into teratomas over four to eight weeks. The teratomas were fixed in 4% paraformaldehyde, transferred to ethanol for gradient dehydration, embedded in paraffin, and sliced into 4 µm sections for hematoxylin and eosin (H&E) staining. All animal experiments were approved by and conducted in accordance with the guidelines of the Animal Care and Use Committee of the Guangzhou Institutes of Biomedicine and Health, Chinese Academy of Sciences.

### Differentiation of iPSCs into neurons

iPSCs were differentiated into neurons using a standard monolayer differentiation protocol with minor modifications, as previously described [20]. Briefly, iPSCs were seeded onto a Matrigel-coated 12-well plate in mTeSR™ Plus medium. Upon reaching 100% confluence, the culture medium was replaced with N2B27 medium, consisting of 50% DMEM/F12 medium (Gibco, Cat# 11320033), 50% neurobasal medium (Gibco, Cat# 21103049), 0.5× N2 supplement (Gibco, Cat# 17502048), 0.5× B27 supplement (Gibco, Cat# 17504044), 1% GlutaMAX™ supplement (Gibco, Cat# 35050061), 1% non-essential amino acids (NEAA) supplement (Gibco, Cat# 11140050), and 5 μg/mL human insulin (Sigma-Aldrich, Cat# I9278), supplemented with 5 μM SB431542 (Selleck Chemicals, Cat# S1067) and 5 μM dorsomorphin (Selleck Chemicals, Cat# S7306) for 8 days. The medium was changed daily. After 8 days, the cells were passaged onto a Matrigel-coated 6-well plate in N2B27 medium, with medium changed every other day. After another 8 days, neural progenitor cells (NPCs) were manually picked and transferred to a low-attachment 6-well plate in N2B27 medium containing 20 ng/mL bFGF (PeproTech, Cat# 100-18B) to expand for 7 days, with medium replacement every other day. For differentiation, NPCs were dissociated into single cells using Accutase and seeded onto poly-L-lysine- and laminin-coated plates at a density of 10,000 cells/cm^2^ in N2B27 medium supplemented with 20 ng/mL GDNF (PeproTech, Cat# 450-10) and 20 ng/mL BDNF (PeproTech, Cat# 450-02). Differentiation took 30 days, during which the medium was replaced every other day. The neurons matured over this period and were collected for further examination.

### Morphometric analysis of neurons

NPCs (Day 23) were dissociated and plated at a density of 45,000 cells per well onto coverslip in a 12-well plate that had been pre-coated with poly-L-lysine at room temperature overnight and then with laminin at 37 °C for at least 1 h for further differentiation. After 7 days of attachment, the neurons were immunostained for TUJ1 and imaged using a confocal laser-scanning microscope (LSM 980, Zeiss). Soma size was defined as the cross-sectional area of the cell body. The number of primary branches was defined as the number of dendritic trees emerging directly from the soma per neuron. The number of branching points was defined as the number of nodes (points at which a dendrite branches into two or more) per neuron. At least 40 randomly selected neurons per group from three independent replicates were analyzed using ImageJ software (National Institutes of Health, NIH).

### Isolation of mitochondria

Mitochondria were isolated using a Mitochondria Isolation Kit (Abcam, Cat# ab110170) according to the manufacturer’s instructions. Briefly, cells were dissociated using Accutase and pelleted by centrifugation at 1000 × *g*. The cell pellet was resuspended in Reagent A to a concentration of 5 mg/mL, incubated on ice for 10 min and then homogenized using a Dounce homogenizer. The homogenate was centrifuged at 1000 × *g* and 4 °C for 10 min. After the first centrifugation, the supernatant was collected for further use. The pellet containing large debris was resuspended in Reagent B, incubated on ice for 10 min, homogenized, and centrifuged at 1000 × *g* and 4 °C for 10 min. The supernatants from both steps were combined and centrifuged at 12,000 × *g* and 4 °C for 15 min to isolate the mitochondrial pellet from the cytosolic fraction. The mitochondrial pellet was resuspended in an appropriate volume of Reagent C and stored at −80 °C for subsequent analysis.

### Quantitative real-time PCR (qRT-PCR)

Total RNA was extracted using the TRIzol reagent (Sigma-Aldrich, Cat# T3934) according to the manufacturer’s instructions. Next, 1 μg of total RNA was reverse transcribed into cDNA using the HiScript III RT SuperMix for qPCR (+gDNA wiper) (Vazyme, Cat# R323-01). Subsequently, the qRT-PCR assay was performed using the ChamQ Universal SYBR qPCR Master Mix (Vazyme, Cat# Q711-02) on a QuantStudio 3 instrument (Thermo Fisher). The forward and reverse primer sequences for *TIMM8A* were 5’-CAGCATTTCATCGAGGTAGAGAC-3’ and 5’-AGCCCGACTGTCCAACTTTG-3’, respectively.

### Immunofluorescence staining and confocal microscopy

Cells grown on Matrigel-coated coverslips were washed with PBS and fixed with 4% paraformaldehyde (PFA) for 30 min at room temperature. After washing with PBS, cells were permeabilized with 0.25% Triton X-100 for 20 min, blocked with 5% bovine serum albumin (BSA) for 1 h, and incubated with primary antibodies at 4 °C overnight. After washing with PBS, cells were incubated with Alexa Fluor 488- or Alexa Fluor 568-conjugated secondary antibodies for 60 min at room temperature. Cells were washed with PBS and mounted using an anti-fade fluorescence mounting medium containing DAPI (Solarbio, Cat# S2110). Fluorescence signals were visualized and captured using a confocal laser-scanning microscope (LSM 980, Zeiss). The primary antibodies used in this study were as follows: anti-NANOG antibody (Cell Signaling Technology, Cat# 3580), anti-OCT4 antibody (Santa Cruz, Cat# sc-5279), anti-SOX2 antibody (R&D Systems, Cat# MAB2018), anti-SSEA4 antibody (Abcam, Cat# ab16287), anti-PAX6 antibody (Biolegend, Cat# PRB-278P), anti-SOX1 antibody (R&D Systems, Cat# AF3369-SP), anti-Nestin antibody (Millipore, Cat# ABD69), anti-TUJ1 antibody (GeneTex, Cat# GTX130245), and anti-MAP2 antibody (Cell Signaling Technology, Cat# 4542).

### Western blot analysis

Western blot analysis was performed according to a standard protocol routinely used in our laboratory [18]. The primary antibodies used in this study were as follows: anti-SOX2 antibody (Cell Signaling Technology, Cat# 2748), anti-PAX6 antibody (Biolegend, Cat# PRB-278P), anti-Nestin antibody (Millipore, Cat# ABD69), anti-TIMM8A antibody (Proteintech, Cat# 11179-1-AP), anti-HSP60 antibody (Proteintech, Cat# 15282-1-AP), anti-TOMM40 antibody (Proteintech, Cat# 18409-1-AP), anti-TIMM23 antibody (Proteintech, Cat# 11123-1-AP), anti-COX4 antibody (Proteintech, Cat# 11242-1-AP), anti- Cytochrome c antibody (Proteintech, Cat# 10993-1-AP), anti-cleaved caspase-3 antibody (Cell Signaling Technology, Cat# 9661), anti-BCL-2 antibody (ABclonal, Cat# A19693), anti-BAX antibody (Proteintech, Cat# 60267-1-Ig), anti-CHCHD2 antibody (Proteintech, Cat# 19424-1-AP), anti-Flag antibody (Proteintech, Cat# 66008-4-Ig), and anti-Beta Tubulin antibody (Proteintech, Cat# 66240-1-Ig). The secondary antibodies were HRP-conjugated Goat Anti-Rabbit IgG(H + L) antibody (Proteintech, Cat# SA00001-2) and HRP-conjugated Goat Anti-Mouse IgG(H + L) antibody (Proteintech, Cat# SA00001-1). Signals were detected using an automated chemiluminescence imaging system (Tanon).

### ATP measurement

The ATP concentration in neurons was measured using the CellTiter-Glo Luminescent Cell Viability Assay Kit (Promega, Cat# G7570) according to the manufacturer’s protocol. Briefly, neurons were harvested and seeded onto a clear-bottom, black-walled 96-well microplate at a density of 30,000 cells per well and cultured overnight before measurement. After equilibration at room temperature for approximately 30 min, an equal volume of CellTiter-Glo® Reagent was added to each well and mixed on an orbital shaker for 2 min to induce cell lysis. Luminescence intensity was measured after a 10-min incubation period.

### Reactive oxygen species (ROS) measurement

The ROS concentration was measured using the DCFDA/H2DCFDA - Cellular ROS Assay Kit (Abcam, Cat# ab113851) according to the manufacturer’s protocol. Briefly, neurons were harvested and seeded onto a clear-bottom, black-walled 96-well microplate at a density of 25,000 cells per well and cultured overnight before measurement. Cells were then incubated with DCFDA solution (20 μM final concentration) for 45 min at 37 °C in the dark. Fluorescence was measured immediately using a plate reader at excitation/emission (Ex/Em) wavelengths of 485/535 nm.

### Activity analysis of mitochondrial complex IV

The activity of mitochondrial complex IV was measured using the Cell Mitochondrial Complex IV (Cytochrome C Oxidase) Activity Assay Kit (Elabscience, Cat# E-BC-K837-M) according to the manufacturer’s protocol.

### Construction of stable cell lines overexpressing CHCHD2

To overexpress human CHCHD2 in iPSCs, cells were transduced with lentivirus encoding human CHCHD2 or an empty vector control. Human CHCHD2 cDNA was cloned into the pKD-Flag-IRES2-Puro transfer plasmid using BamHI and EcoRI restriction sites to generate C-terminally Flag-tagged CHCHD2. A total of 2.5 μg of the transfer plasmid, 1.5 μg of the envelope plasmid (pMD2.G), and 1 μg of the packaging plasmid (psPAX2) were co-transfected into 293 T cells using Lipofectamine™ 3000 Transfection Reagent (Invitrogen, Cat# L3000015). After 12 h, the culture medium containing the transfection reagent was replaced with fresh DMEM/F12 medium. Culture supernatants containing lentivirus were collected at 48 h and 72 h post-transfection and centrifuged at 1,000 rpm for 5 min to remove 293T cells. CTRL- and MTS-iPSCs were seeded in 12-well plates and cultured in a mixture of 875 μL mTeSR™ Plus medium and 125 μL lentivirus supernatant. The culture medium was replaced with fresh mTeSR™ Plus medium after 12 h. After 48 h of infection, the culture medium was replaced daily with mTeSR™ Plus medium containing 1 μg/mL puromycin to eliminate uninfected cells, until no cells survived in the negative control wells. Puromycin-resistant cells were expanded and validated by Western blot analysis.

### Transmission electron microscopy

Cells were collected and centrifuged at 1000 rpm for 5 min. The medium was discarded, and 1 mL of 2.5% glutaraldehyde was added and incubated for 1 h at room temperature. Cell clumps were further fixed by immersion in 2.5% glutaraldehyde for at least 12 h, rinsed with PBS, and post-fixed with 1% osmium tetroxide for 1 h at 4 °C. The samples were rinsed three times with distilled water (ddH_2_O) and pre-stained with 1% uranyl acetate for 1 h at 37 °C. The samples were dehydrated through a graded series of acetone (50%, 70%, 80%, 90%, and 100%) for 5 min each to ensure complete dehydration. After embedding, the samples were sectioned and imaged using a transmission electron microscope (HITACHI-H7500, Hitachi). The aspect ratio was defined as the ratio of the major axis to the minor axis. The form factor was calculated as (perimeter^2^)/(4π × area). At least 100 mitochondria per group, randomly selected from three independent replicates at a magnification of 15,000×, were analyzed using ImageJ software (NIH).

### Statistical analysis

The sample size was not predetermined by statistical methods. The experiments were not randomized, and investigators were not blinded during the experiment. All statistical analyses were performed using GraphPad Prism 9 software (GraphPad Software, USA). One-way or two-way ANOVA was used for comparisons among multiple groups, followed by Tukey’s post hoc test for specific group comparisons. A two-tailed unpaired Student’s *t* test was used for comparisons between two groups. Data are presented as mean ± standard deviation (SD), and *p* < 0.05 is considered statistically significant. The relevant figure legends include details of the statistical tests used, exact *p*-values, and the number of independent replicates.

## Results

### Construction of iPSCs with a novel mutation in *TIMM8A* from an MTS patient

Our patient, a 10-year-old boy, developed progressive bilateral sensorineural hearing loss from early childhood, accompanied by severe dystonia starting at the age of ten. Genetic analysis indicated that he has a small deletion variation in *TIMM8A* (c.225-229del), a novel, likely pathogenic, hemizygous mutation inherited from his mother. This mutation leads to a frameshift mutation in the TIMM8a protein beginning at the 75^th^ amino acid residue (p.Q75fs95*).

**Fig. 1:**
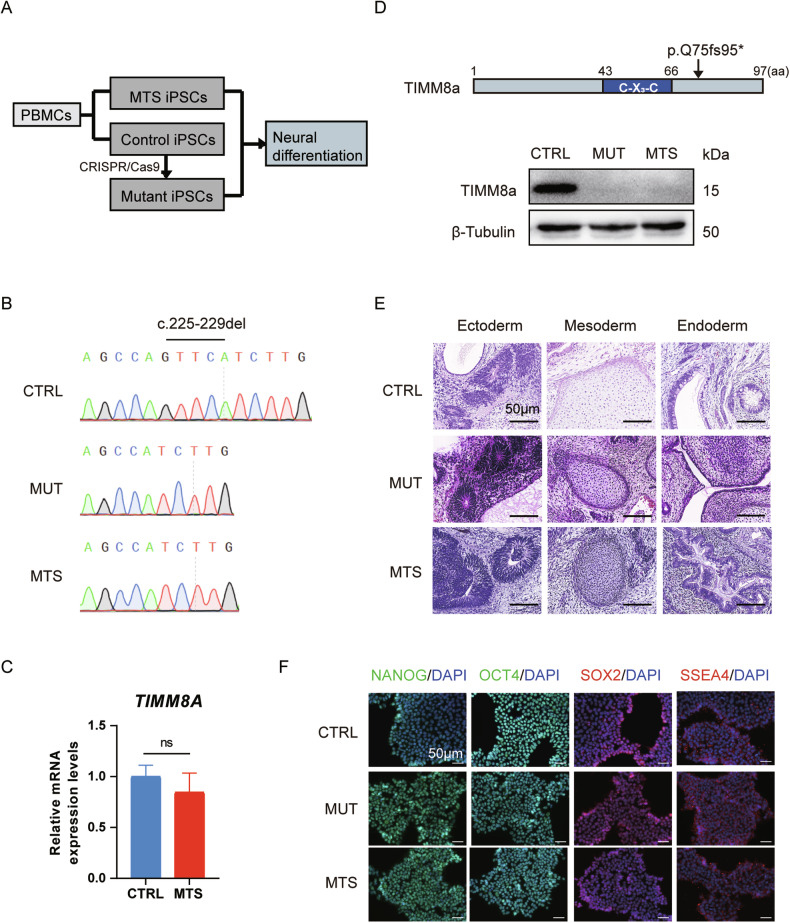
Construction of iPSCs with a novel mutation in *TIMM8A.* **A** Schematic diagram illustrating the generation of three induced pluripotent stem cell (iPSC) lines: MTS-iPSCs and control iPSCs (CTRL-iPSCs) were derived from an MTS patient carrying a novel mutation in *TIMM8A* (c.225-229del) and a healthy individual, respectively, while mutant iPSCs (MUT-iPSCs) were generated from CTRL-iPSCs using CRISPR/Cas9. All iPSCs were reprogrammed from peripheral blood mononuclear cells (PBMCs). **B** Partial Sanger sequencing chromatograms of *TIMM8A* in the CTRL-, MUT-, and MTS-iPSCs, showing the c.225-229del mutation. **C** mRNA levels of *TIMM8A* in CTRL- and MTS-iPSCs. *n* = 3 independent biological replicates. Data are represented as mean ± SD. Significance was determined by two-tailed unpaired Student’s *t* test: ns, not significant. **D** Western blot analysis showing the absence of TIMM8a protein with a frameshift mutation starting at the 75^th^ amino acid residue (p.Q75fs95*), encoded by the mutant *TIMM8A* gene, in MUT- and MTS-iPSCs. **E** Hematoxylin and eosin staining of the three germ layers successfully differentiated from iPSCs in vivo. Scale bar: 50 μm. **F** Immunostaining confirming the generation of CTRL-, MUT-, and MTS-iPSCs using pluripotency markers, including NANOG (green), OCT4 (green), SOX2 (red), and SSEA4 (red). Nuclei were counterstained with DAPI (blue). Scale bar: 50 μm.

To elucidate the pathophysiology of MTS, we established MTS-iPSCs and CTRL-iPSCs using PBMCs from the patient and a healthy individual, respectively (Fig. 1A). To obtain an isogenic iPSC line containing the same mutant *TIMM8A* as our patient, we used the CRISPR/Cas9 to generate MUT-iPSCs based on CTRL-iPSCs (Fig. 1A). The mutation in the *TIMM8A* gene was confirmed in both MUT-iPSCs and MTS-iPSCs (Fig. 1B). Patient-derived iPSCs exhibited normal *TIMM8A* mRNA levels (Fig. 1C). Western blot analysis confirmed the absence of TIMM8a protein in both MUT-iPSCs and MTS-iPSCs but not in CTRL-iPSCs (Fig. 1D). All three iPSC lines were capable of differentiating into the three germ layers in vivo (Fig. 1E). Additionally, they exhibited positive staining for pluripotency markers in vitro, including NANOG, OCT4, SOX2 and SSEA4 (Fig. 1F). These data confirmed the successful construction of MTS-, MUT-, and CTRL-iPSCs.

### *TIMM8A* mutation impairs neuronal differentiation in iPSCs

**Fig. 2:**
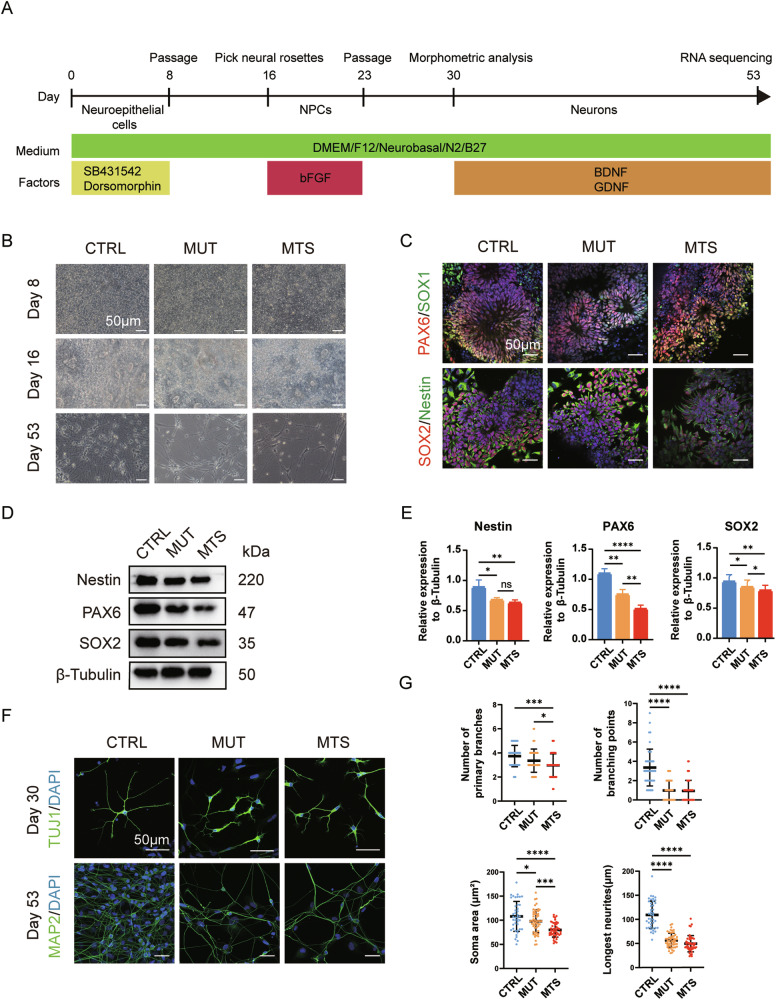
Defects in neuronal differentiation of iPSCs due to mutation in *TIMM8A.* **A** Schematic diagram of the neuronal differentiation strategy: iPSCs were differentiated sequentially into neuroepithelial cells, neural progenitor cells (NPCs), and finally mature neurons over a period of 53 days. **B** Morphology of CTRL-, MUT-, and MTS-derived cells at day 8 (neuroepithelial cells), day 16 (NPCs), and day 53 (mature neurons) during differentiation. Scale bar: 50 μm. **C** Generations of CTRL-, MUT- and MTS-NPCs was confirmed by immunostaining with NPC markers, including PAX6 (red), SOX1 (green), SOX2 (red), and Nestin (green) in rosette-like cells at day 16. Nuclei were stained with DAPI (blue). Scale bar: 50 μm. **D**, **E** Expression levels of Nestin, PAX6, and SOX2 in CTRL-, MUT-, and MTS-NPCs were analyzed by Western blot. Relative protein levels were quantified and normalized to β-Tubulin as a loading control. *n* = 3 independent biological replicates. Data are represented as mean ± SD. Significance was determined by one-way ANOVA with Tukey’s post hoc test: ns, not significant; *p < 0.05; **p < 0.01; ****p < 0.0001. **F** Generation of early neurons at day 30 and mature neurons at day 53 was confirmed by immunostaining with the early neuronal marker TUJ1 (green) and the mature neuronal marker MAP2 (green), respectively. Nuclei were stained with DAPI (blue). Scale bar: 50 μm. **G** Quantification of branch numbers, soma size, and neurite length in early neurons at day 30. *n* = 3 independent biological replicates (42 neurons for CTRL, 53 for MUT, and 46 for MTS). Data are represented as mean ± SD. Significance was determined by one-way ANOVA with Tukey’s post hoc test: *p < 0.05; ***p < 0.001; ****p < 0.0001.

Next, we differentiated MTS-, MUT-, and CTRL-iPSCs into neurons following the protocol outlined in Fig. 2A. Upon application of dual SMAD inhibition, iPSCs were differentiated into NPCs, with the emergence of neural rosettes at day 16, and finally developed into mature neurons at day 53 (Fig. 2B). To confirm differentiation toward the neural lineage, we first measured the expression of markers of NPCs, including PAX6, SOX1, SOX2, and Nestin. Immunocytochemistry analysis revealed positive expression of PAX6, SOX1, SOX2, and Nestin in NPCs after 16 days of differentiation (Fig. 2C). We then performed Western blot analysis to compare the relative expression levels of Nestin, PAX6, and SOX2 among the three groups. The results revealed a significant reduction in both MUT-NPCs and MTS-NPCs compared to CTRL-NPCs, with greater reductions in PAX6 and SOX2 expression observed in MTS-NPCs (Fig. 2D, E). These results suggest that TIMM8a loss-of-function impairs iPSC differentiation into NPCs.

Subsequently, we further differentiated NPCs into neurons through an additional 30-day differentiation period. Microscopic observation revealed a noticeable decrease in cell numbers in both MTS- and MUT-neurons (Fig. 2B, lowest panel). Immunocytochemistry analysis revealed morphometric phenotypes in early neurons (day 30) labeled with TUJ1 and in mature neurons (day 53) labeled with MAP2. As shown in Fig. 2F, significant neuronal developmental defects were observed at both early and mature stages, characterized by fewer branches, smaller somata, and shorter neurites in early neurons (Fig. 2G). Thus, TIMM8a loss-of-function may contribute to defects in neurite elongation.

### *TIMM8A* mutation causes mitochondrial dysfunction in iPSC-derived neurons

**Fig. 3:**
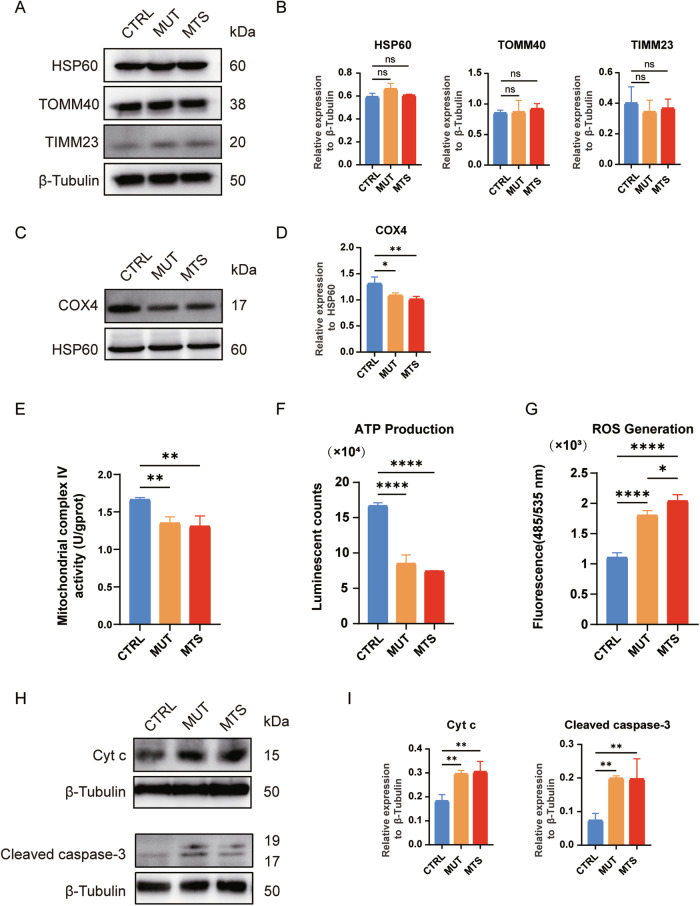
Dysfunction of mitochondria in iPSC-derived neurons due to mutation in *TIMM8A.* **A**, **B** No differences in HSP60, TOMM40, and TIMM23 levels were observed among CTRL-, MUT-, and MTS-neurons by Western blot. Relative protein levels were quantified and normalized to β-Tubulin as a loading control. *n* = 3 independent biological replicates. **C**, **D**. Western blot analysis showed decreased levels of COX4 in mitochondrial complex IV in MUT- and MTS-neurons. Relative levels of COX4 were quantified and normalized to HSP60 as a loading control. *n* = 3 independent biological replicates. **E** The activity of mitochondrial complex IV was decreased in MUT- and MTS-neurons compared to CTRL-neurons. *n* = 3 independent biological replicates. **F** ATP production was decreased in MUT- and MTS-neurons compared to CTRL-neurons. *n* = 3 independent biological replicates. **G** ROS levels were increased in MUT- and MTS-neurons compared to CTRL-neurons. *n* = 3 independent biological replicates. **H**, **I** Levels of Cyt c and cleaved caspase-3 in CTRL-, MUT-, and MTS-neurons after treatment with 100 µM TBHP for 24 h were analyzed by Western blot. Relative protein levels of Cyt c and cleaved caspase-3 in the cytosol were quantified and normalized to β-Tubulin as a loading control. *n* = 3 independent biological replicates. Data are represented as mean ± SD. Significance was determined by one-way ANOVA with Tukey’s post hoc test: ns, not significant; *p < 0.05; **p < 0.01; ****p < 0.0001 (applied to panels **B**, **D**–**G**, and **I**).

Since Tim8p, the yeast homolog of TIMM8a, forms a hexamer with Tim13p to participate in membrane protein transport, we first analyzed the levels of several mitochondrial proteins in neurons, including heat-shock protein 60 (HSP60), translocase of outer mitochondrial membrane 40 (TOMM40), and translocase of inner mitochondrial membrane 23 (TIMM23). No significant changes were observed among MUT-, MTS-, and CTRL-neurons (Fig. 3A, B). Next, we examined the effect of *TIMM8A* mutation on mitochondrial complex IV, as *TIMM8A* silencing has been reported to be associated with mitochondrial complex IV dysfunction [16, 17]. Consistent with previous studies, we observed a significant reduction in cytochrome c oxidase subunit 4 (COX4) levels in mitochondria isolated from MTS- and MUT-neurons (Fig. 3C, D). In addition, a significant defect in complex IV activity was observed in both MUT- and MTS-neurons (Fig. 3E).

Next, we measured energy production and ROS generation in the three groups. ATP levels in MUT- and MTS-neurons were significantly reduced compared to CTRL-neurons (Fig. 3F). Moreover, MUT- and MTS-neurons exhibited significantly elevated ROS levels compared to CTRL-neurons, with a more severe phenotype observed in MTS-neurons (Fig. 3G), indicating increased oxidative stress due to TIMM8a deficiency.

To determine whether TIMM8a deficiency, combined with enhanced oxidative stress, leads to increased neuronal apoptosis, we treated the neurons for 24 h with 100 µM tert-Butyl Hydroperoxide (TBHP), an oxidative stress-inducing agent, and measured the levels of cytochrome c (Cyt c) and cleaved caspase-3 in the cytosol. As shown in Fig. 3H and I, elevated levels of Cyt c and cleaved caspase-3 were observed in MUT- and MTS-neurons compared to CTRL-neurons. Thus, TIMM8a deficiency increased apoptosis sensitivity, priming cells for death under oxidative stress.

### RNA sequencing reveals differential gene expression in iPSC-derived neurons

**Fig. 4:**
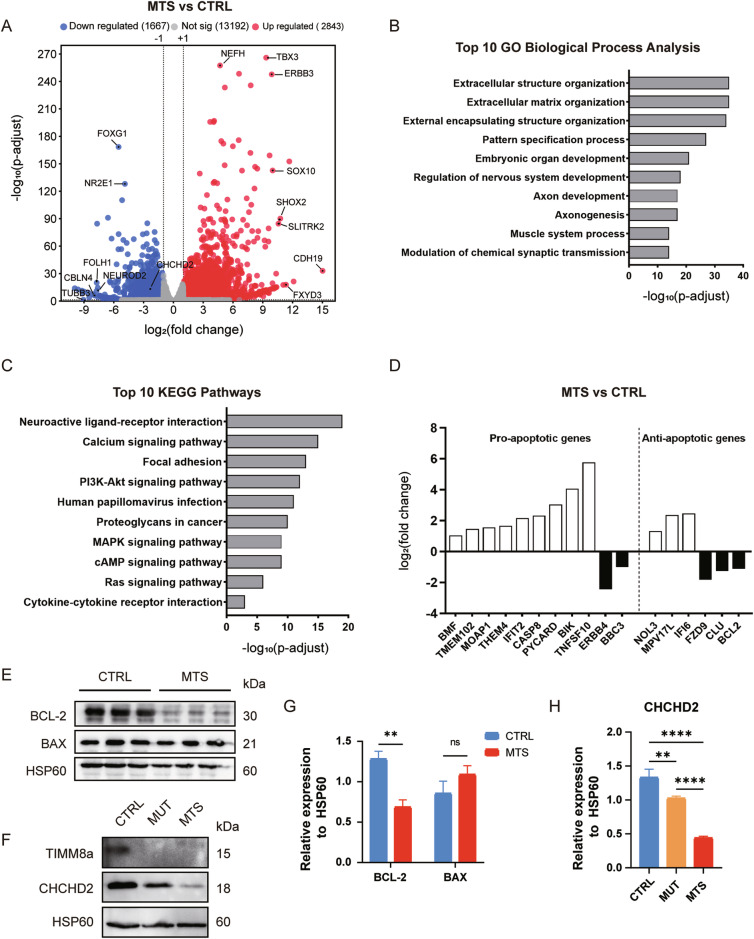
Altered gene expression in iPSC-derived neurons revealed by RNA sequencing. **A** A total of 2,843 genes were significantly upregulated, and 1,667 genes were significantly downregulated in MTS-neurons compared to CTRL-neurons (adjusted *p* < 0.05 and |log2(fold change)| > 1). Top altered genes, including *CHCHD2*, are labeled. **B** Top 10 altered biological processes in MTS-neurons compared to CTRL-neurons, identified by Gene Ontology (GO) enrichment analysis (adjusted *p* < 0.05). **C** Top 10 altered Kyoto Encyclopedia of Genes and Genomes (KEGG) pathways in MTS-neurons compared to CTRL-neurons (adjusted *p* < 0.05). **D** Representative differentially expressed genes involved in mitochondria-related apoptosis. **E** Western blot analysis of the anti-apoptotic protein BCL-2 and the pro-apoptotic protein BAX in isolated mitochondrial lysates from CTRL- and MTS-neurons. **F** Western blot analysis of CHCHD2 in isolated mitochondrial lysates from CTRL-, MUT-, and MTS-neurons. **G** Expression levels of BCL-2 and BAX were quantified and normalized to HSP60 as a loading control. *n* = 3 independent biological replicates. Data are represented as mean ± SD. Significance was determined by two-tailed unpaired Student’s *t* test: ns, not significant; **p < 0.01. **H** CHCHD2 expression levels were quantified and normalized to HSP60 as a loading control. *n* = 3 independent biological replicates. Data are represented as mean ± SD. Significance was determined by one-way ANOVA with Tukey’s post hoc test: **p < 0.01; ****p < 0.0001.

To elucidate the molecular mechanisms underlying the defects in MTS-neurons, we performed RNA sequencing (RNA-seq) using neurons at day 53 differentiated from three independent iPSC batches. Among the 4510 differentially expressed genes (DEGs), 2843 genes were significantly upregulated, and 1667 genes were significantly downregulated in MTS-neurons compared to CTRL-neurons (Fig. 4A and supplementary Table S1), with an adjusted *p*-value < 0.05 and an absolute log_2_(fold change) > 1. Gene ontology (GO) enrichment analysis and Kyoto Encyclopedia of Genes and Genomes (KEGG) pathway analysis were then performed to identify enriched biological functions and pathways.

GO enrichment analysis for biological processes revealed approximately 1836 pathways significantly altered in MTS-neurons compared to controls (adjusted *p*-value < 0.05), with the top 10 pathways listed in Fig. 4B and supplementary Table S2. Development-related terms were highly enriched among these pathways, including pattern specification processes, embryonic organ development, and nervous system development, particularly those associated with axonogenesis and synaptic activity. KEGG pathway analysis identified 111 significantly enriched pathways (adjusted *p*-value < 0.05), with the top 10 pathways associated with neuroactive ligand-receptor interactions and the PI3K-Akt signaling pathway (Fig. 4C and supplementary Table S3).

Notably, 20 GO terms (adjusted *p*-value < 0.05) were enriched in the regulation of apoptosis, with 76 genes involved in the neuronal apoptotic process. Since TIMM8a is a mitochondrial protein, GO enrichment analysis identified 17 genes involved in modulating mitochondrial apoptotic changes (Fig. 4D and supplementary Table S4). TIMM8a dysfunction resulted in a more than 2-fold increase in the expression of positive regulators of mitochondrial apoptosis, including *BMF*, *MOAP1*, *PYCARD*, *CASP8*, and *BIK*. In contrast, the expression of anti-apoptotic genes, such as *FZD9*, *CLU*, and *BCL-2*, was reduced by more than 2-fold. Western blot analysis confirmed that BCL-2 levels were significantly reduced in MTS-neurons compared to controls (*p* < 0.01), with no increase in BAX levels (Fig. 4E, G). Consistent with the elevated ROS levels observed in MTS-neurons, 118 genes were enriched in the GO term “response to oxidative stress.” Among these genes, *CHCHD2*, encoding a mitochondrial IMS protein that protects against oxidative stress in neuronal cells [21], showed a more than 4-fold decrease in expression in the MTS group, as revealed by RNA-seq. A significant reduction in CHCHD2 protein levels was confirmed in both MTS- and MUT-neurons by Western blot analysis (Fig. 4F, H).

### Overexpression of CHCHD2 rescues mitochondrial dysfunction and improves neurogenesis

**Fig. 5:**
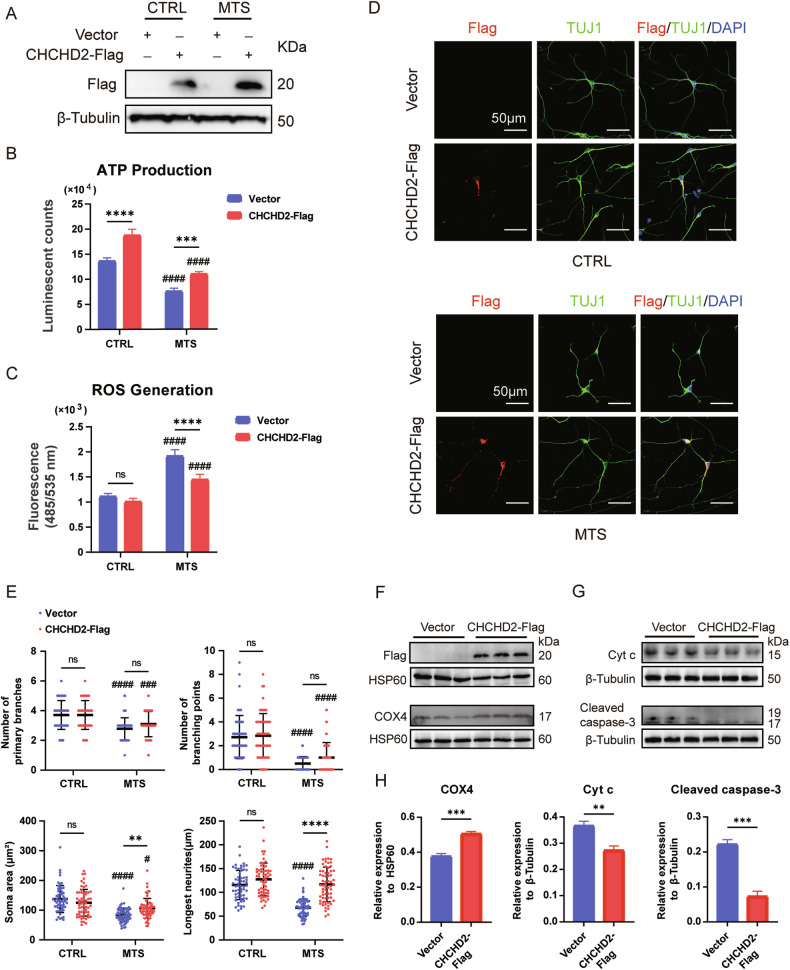
Rescue of mitochondrial dysfunction and neurogenesis defects by CHCHD2 overexpression in iPSC-derived neurons. **A** Overexpression of CHCHD2-Flag was confirmed by Western blot in CTRL- and MTS-iPSCs. “Vector” represents empty lentivirus, and “CHCHD2-Flag” represents lentivirus expressing CHCHD2-Flag. **B** ATP synthesis was significantly decreased in MTS-neurons compared to CTRL-neurons. Overexpression of CHCHD2 increased ATP production in both CTRL- and MTS-neurons. *n* = 3 independent biological replicates. **C** ROS generation was significantly increased in MTS-neurons compared to CTRL-neurons. Overexpression of CHCHD2 decreased ROS generation in MTS-neurons. *n* = 4 independent biological replicates. **D** Immunostaining for the neuronal marker TUJ1 (green) and Flag tag (red) in CTRL- and MTS-neurons at day 30. Nuclei were stained with DAPI (blue). Scale bar: 50 μm. **E** Quantification of branch numbers, soma size, and neurite length in neurons at day 30. *n* = 3 independent biological replicates (58 neurons for Vector group and 60 for CHCHD2-Flag group in CTRL-neurons; 64 neurons for Vector group and 64 for CHCHD2-Flag group in MTS-neurons). **F** Western blot analysis of COX4 levels in mitochondria isolated from MTS-neurons in the Vector and CHCHD2-Flag groups. **G** Western blot analysis of Cyt c and cleaved caspase-3 levels in the cytosol of MTS-neurons after treatment with 100 µM TBHP for 24 h in the Vector and CHCHD2-Flag groups. **H** Expression levels of COX4 were quantified and normalized to HSP60, and levels of Cyt c and cleaved caspase-3 were quantified and normalized to β-Tubulin. *n* = 3 independent biological replicates. Statistical significance was determined as follows: For panels **B**, **C**, and **E**, two-way ANOVA followed by Tukey’s post hoc test was used. Data are represented as mean ± SD: ns, not significant; **p < 0.01; ***p < 0.001; ****p < 0.0001 for shown comparisons; #*p* < 0.05; ###*p* < 0.001; ####*p* < 0.0001 relative to the corresponding CTRL group. For panel **H** a two-tailed unpaired Student’s *t* test was used. Data are represented as mean ± SD: **p < 0.01; ***p < 0.001.

CHCHD2 has been reported as an interacting partner of TIMM8a in neuronal cells [16]. Therefore, we overexpressed CHCHD2 to determine whether it could rescue the phenotypes of MTS-neurons. Interestingly, overexpression of Flag-tagged human CHCHD2 in MTS-iPSCs and CTRL-iPSCs via lentiviral transduction (Fig. 5A) enhanced ATP production in neurons from both groups (Fig. 5B) and reduced ROS levels in MTS-neurons (Fig. 5C). To further investigate the role of CHCHD2 in early neuronal differentiation, we analyzed neuronal somata, branches, and neurites. No significant changes were observed between the vector and CHCHD2-Flag groups in CTRL-neurons (Fig. 5D, E). However, in MTS-neurons, a slight increase in soma size was observed in the CHCHD2-Flag group compared to the vector group. Most importantly, CHCHD2-Flag overexpression effectively elongated neurites (Fig. 5D, E). Notably, COX4 levels were significantly upregulated in mitochondrial complex IV of MTS-neurons overexpressing CHCHD2-Flag compared to the vector group (Fig. 5F, H). In addition, reduced levels of Cyt c leakage from mitochondria and cleaved caspase-3 were observed in MTS-neurons overexpressing CHCHD2-Flag compared to the vector group following TBHP treatment (Fig. 5G, H). Taken together, these findings suggest that CHCHD2 may act as a downstream effector of TIMM8a, regulating neurogenesis through the maintenance of mitochondrial homeostasis.

### Overexpression of CHCHD2 rescues *TIMM8A* mutation-induced mitochondrial morphology defects

**Fig. 6:**
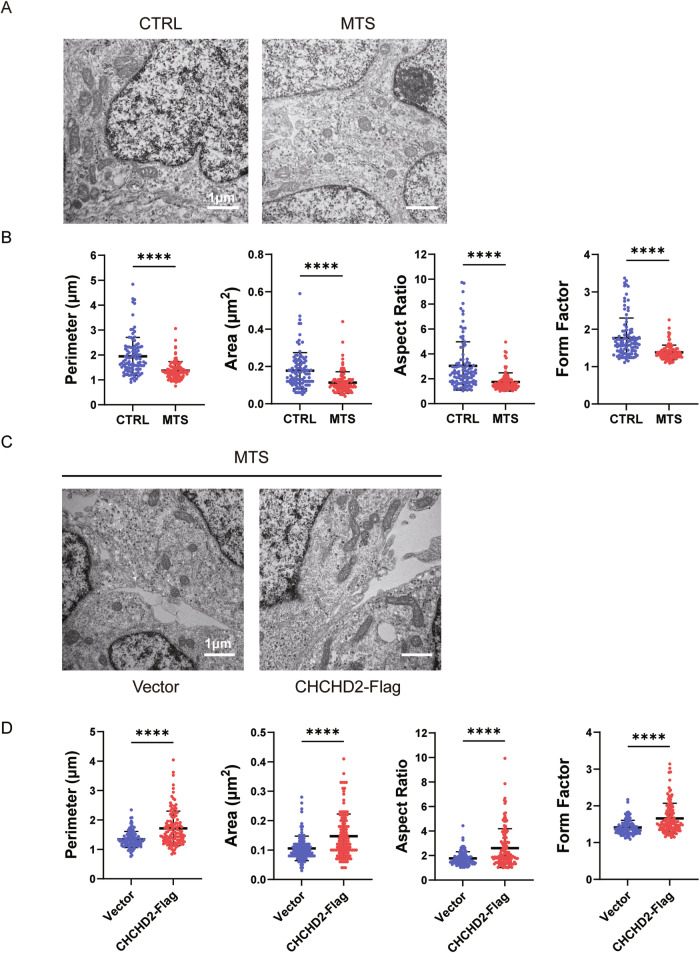
CHCHD2 overexpression rescues mitochondrial morphology defects in iPSC-derived neurons. **A**, **B** Mitochondrial morphology in CTRL- and MTS-neurons at day 30, as revealed by transmission electron microscopy (TEM). Scale bar: 1 μm. *n* = 3 independent biological replicates. Quantification of mitochondrial perimeter, area, aspect ratio, and form factor was performed (103 mitochondria for CTRL group; 102 for MTS group). **C**, **D** Mitochondrial morphology in the Vector group and CHCHD2-Flag group of MTS-neurons at day 30, as revealed by TEM. Scale bar: 1 μm. *n* = 3 independent biological replicates. Quantification of mitochondrial perimeter, area, aspect ratio, and form factor was performed (107 mitochondria for Vector group; 124 for CHCHD2-Flag group). Data are represented as mean ± SD. Significance was determined by two-tailed unpaired Student’s *t* test: **p < 0.01; ****p < 0.0001.

Mitochondrial morphology was analyzed in early neurons (day 30) using transmission electron microscopy (TEM). Although mitochondria in MTS-neurons exhibited well-defined cristae structures, the *TIMM8A* mutation induced significant mitochondrial fragmentation, as evidenced by reductions in perimeter, area, aspect ratio, and form factor compared to the control group (Fig. 6A, B). Importantly, overexpression of human CHCHD2 rescued these morphological alterations in MTS-neurons (Fig. 6C, D).

## Discussion

Our study identified a novel pathogenic mutation in the *TIMM8A* gene (c.225-229del, p.Q75fs95*) in an MTS patient and successfully established MTS-iPSCs. Using CRISPR/Cas9, we generated MUT-iPSCs from CTRL-iPSCs, introducing the same *TIMM8A* mutation found in MTS-iPSCs. Both MTS-iPSCs and MUT-iPSCs exhibited defects in neuronal differentiation characterized by smaller somata, fewer branches, and shorter neurites, along with increased sensitivity to apoptosis under oxidative stress. Importantly, dysfunction of mitochondrial oxidative phosphorylation (OXPHOS) complexes was evidenced by decreased levels of COX4 and impaired complex IV activity, accompanied by reduced ATP synthesis and increased ROS levels in both MTS-neurons and MUT-neurons. RNA-seq revealed a significant downregulation of *CHCHD2* in MTS-neurons, and Western blot analysis confirmed a significant reduction in levels of CHCHD2, a mitochondrial IMS protein, in both MTS-neurons and MUT-neurons. Furthermore, overexpression of CHCHD2 in MTS-neurons protected against mitochondrial dysfunction and partially rescued neurogenesis defects, suggesting that CHCHD2 may act as a downstream effector of TIMM8a.

The novel mutation in the *TIMM8A* gene identified in this study leads to mitochondrial dysfunction and neuronal apoptosis due to the loss of functional TIMM8a protein. The MTS patient exhibited progressive bilateral sensorineural hearing loss and severe dystonia from childhood. Molecular analysis identified a novel mutation in the *TIMM8A* gene, resulting in a C-terminal frameshift mutation starting at the 75^th^ amino acid (c.225-229del, p.Q75fs95*). Although patient-derived iPSCs exhibited normal *TIMM8A* mRNA levels (Fig. 1C), TIMM8a protein was absent (Fig. 1D), suggesting that the mutation may lead to mRNA decay or the production of an unstable TIMM8a protein. MTS is a progressive neurodegenerative disorder, as supported by anatomical studies [5–8]. Our findings revealed defects in the differentiation of MTS- and MUT-iPSCs into neurons (Fig. 2F, G), accompanied by reduced mitochondrial complex IV activity (Fig. 3C–E), decreased ATP production, increased ROS generation (Fig. 3F, G), and heightened sensitivity to apoptosis (Fig. 3H, I), recapitulating the pathological phenotypes of MTS.

Another significant finding of this study is that the absence of TIMM8a leads to reduced COX4 levels and impaired mitochondrial complex IV activity in iPSC-derived neurons. This observation is consistent with the findings of Kang et al., who demonstrated that TIMM8a deficiency disrupted mitochondrial complex IV assembly in SH-SY5Y cells [17]. Consequently, dysfunction of OXPHOS complexes resulted in decreased ATP synthesis and increased ROS generation. Several studies have investigated the role of TIMM8a. Given that its yeast homolog, Tim8p, is involved in Tim23p transport, early studies primarily focused on its role in protein transport. However, the role of TIMM8a in protein transport remains controversial [10–12]. In our study, we found that TIMM23 and TOMM40 levels did not differ significantly among MTS-, MUT-, and CTRL-neurons (Fig. 3A, B). Other studies have reported mitochondrial elongation in fibroblasts from MTS patients with p.C66W and p.Met1Leu mutations [14, 15]. However, MTS-neurons in this study exhibited shortened mitochondria, consistent with observations in hippocampal neurons from previously reported animal model of MTS [19]. The role of TIMM8a may vary between neuronal and non-neuronal cells [17]. Therefore, further studies on neuronal cells are necessary to elucidate the relationship between TIMM8a mutations and mitochondrial dysfunction in MTS.

Notably, we identified CHCHD2, a mitochondrial IMS protein, which was decreased to 33% and 76.9% in MTS- and MUT-neurons, respectively, compared to controls (Fig. 4F, H). Given the critical role of CHCHD2 in mitochondrial function, its reduction may contribute to the observed phenotypes in MTS- and MUT-neurons. CHCHD2 has been reported to regulate mitochondrial electron transport and energy metabolism, maintain mitochondrial crista structure [22], modulate mitochondrial dynamics [23, 24], inhibit apoptosis [25], protect against oxidative stress [26], and respond to hypoxia [27]. In our study, we detected decreased ATP levels, increased ROS levels, and elevated cytosolic levels of Cyt c and cleaved caspase-3 under oxidative stress in MUT- and MTS-neurons compared to controls (Fig. 3F–I), which may be due to downregulation of CHCHD2. Moreover, the fragmented mitochondria observed in MTS-neurons (Fig. 6A, B) are consistent with findings by Liu et al., who linked fragmented mitochondria in *Chchd2* mutant flies to the degradation of Optic atrophy 1 [23]. The significant decline in BCL-2 suggests increased apoptotic vulnerability in cells lacking CHCHD2 (Fig. 4E, G). The phenotypes observed in MTS-neurons closely resemble those reported in CHCHD2 loss-of-function studies [28–31]. Indeed, previous studies have identified CHCHD2 as an interacting partner of TIMM8a through crosslinking and immunoprecipitation in SH-SY5Y cells [16]. Our findings further suggest that CHCHD2 may act as a downstream mediator of TIMM8a, playing a role in the pathogenic pathway of MTS.

In addition, we found that increasing CHCHD2 expression partially alleviated the neuronal defects caused by the dysfunctional TIMM8a, potentially through the improvement of mitochondrial function. This finding is consistent with previous reports demonstrating CHCHD2-mediated rescue of mitochondrial function [26, 32]. Although MTS-neurons overexpressing CHCHD2 did not fully restore branch complexity to the levels observed in CTRL-neurons, their neurites extended significantly farther from the soma compared to the vector control group (Fig. 5D, E). Notably, compensatory CHCHD2 expression in MTS-neurons upregulated levels of COX4 in complex IV (Fig. 5F, H). The increased COX4 levels may contribute to improved ATP synthesis (Fig. 5B), reduced ROS generation (Fig. 5C), and decreased oxidative stress-induced apoptosis (Fig. 5G, H). Based on these data, we hypothesize that the TIMM8a deficiency leads to downregulation of CHCHD2 and reduced complex IV activity/protein levels. This results in mitochondrial dysfunction, characterized by decreased energy production, elevated oxidative stress, and increased susceptibility to apoptosis, ultimately leading to morphological and functional degeneration in neurons. Our findings suggest that CHCHD2 overexpression may serve as a potential therapeutic strategy for this disorder.

In conclusion, we established MTS patient-derived and CRISPR/Cas9-edited iPSCs, differentiated them into neurons, and demonstrated that the TIMM8a-CHCHD2 pathway is essential for maintaining mitochondrial homeostasis and promoting neuronal development. Future studies should focus on gene rescue approaches in MTS patient-derived iPSCs, and further investigate the molecular mechanisms underlying the interaction between TIMM8a and CHCHD2.

## Supplementary information

Table S1. Differentially expressed genes between the two groups related to Figure 4A.

Table S2. 1836 Gene ontology biological process related to Figure 4B

Table S3. 111 KEGG pathways related to Figure 4C

Table S4. Differentially expressed genes related to apoptotic mitochondrial changes related to Figure 4D.

Images of full and uncropped Western blots

Legends for supplementary materials

## Data Availability

The data supporting the conclusions of this article are included in the manuscript and its supplementary files. The deposited data of RNA-seq is submitted to GEO repository with GEO accession number: GSE284292.
