# Supplementary material for: CHCHD2 rescues the mitochondrial dysfunction in iPSC-derived neurons from patient with Mohr-Tranebjaerg syndrome
Source: Cell Death Dis. 2025 Mar 12;16(1):173. doi: 10.1038/s41419-025-07472-9 (PMC11903874; doi:10.1038/s41419-025-07472-9)
Supplement: Supplementary file 5 — Images of full and uncropped Western blots [file 41419_2025_7472_MOESM5_ESM.pdf]

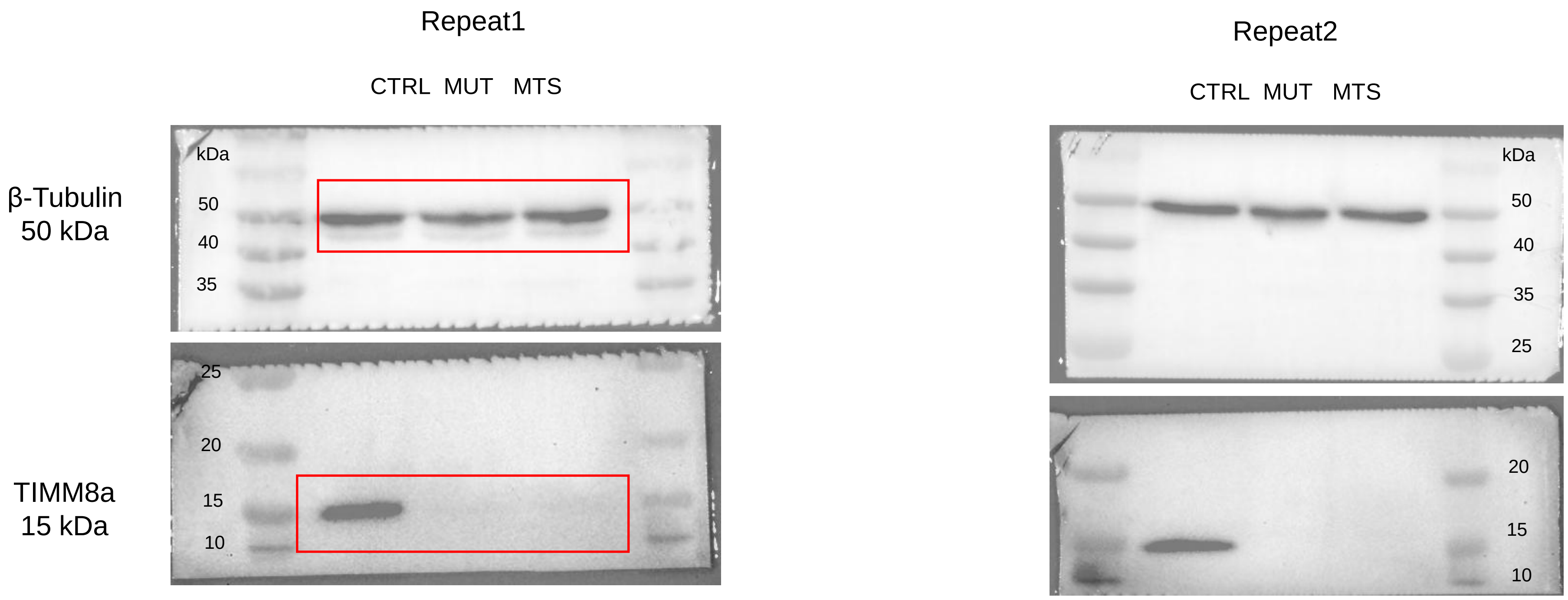

Supplementary Figure 1. Original western blotting images for Figure 1D

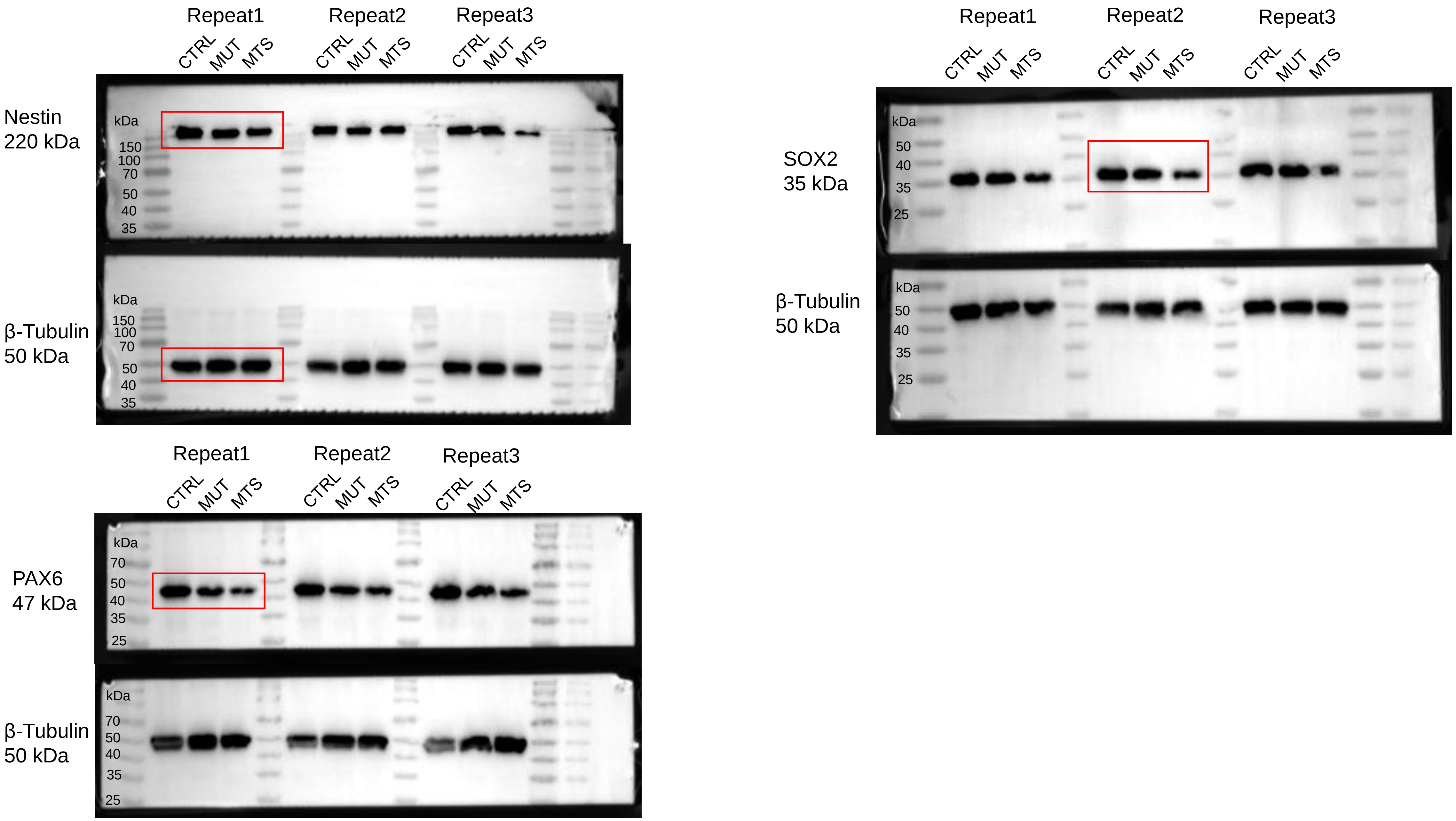

Supplementary Figure 2. Original western blotting images for Figure 2D

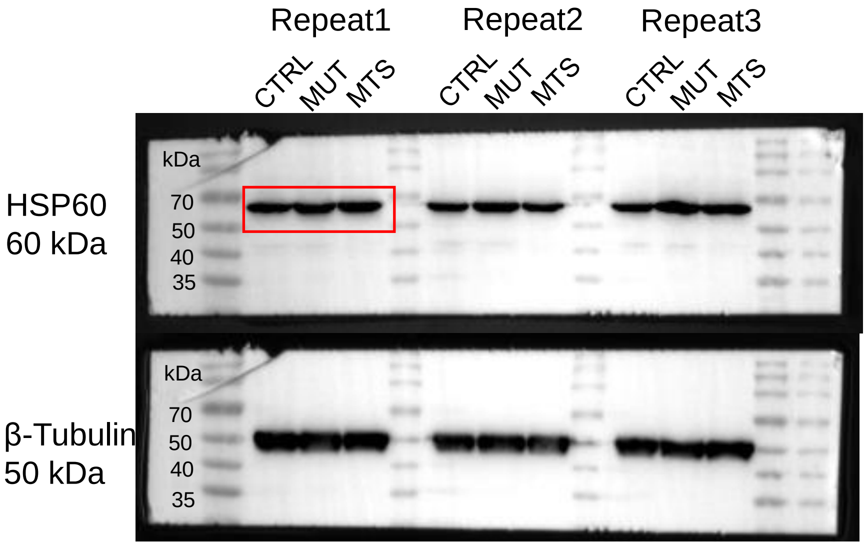

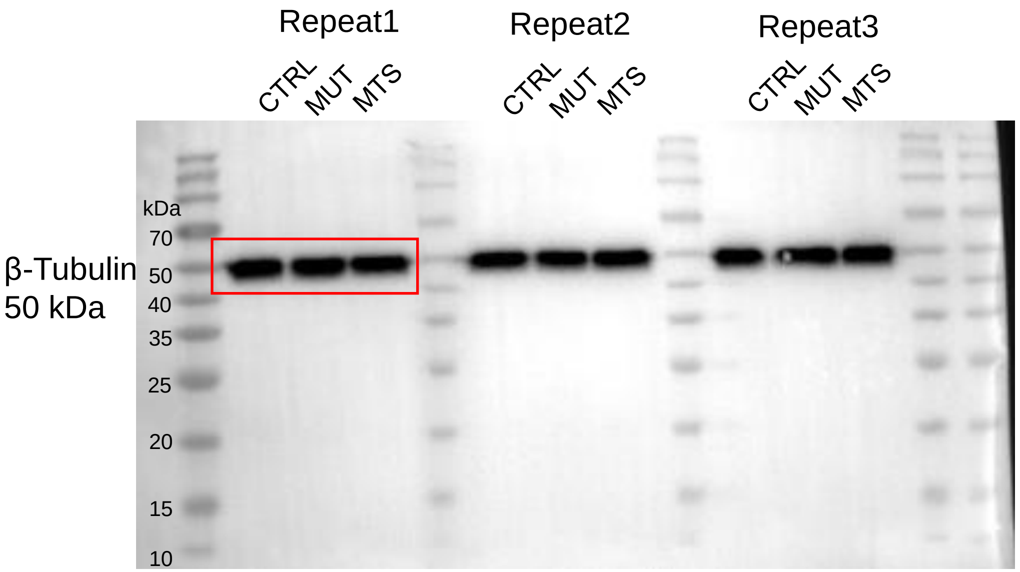

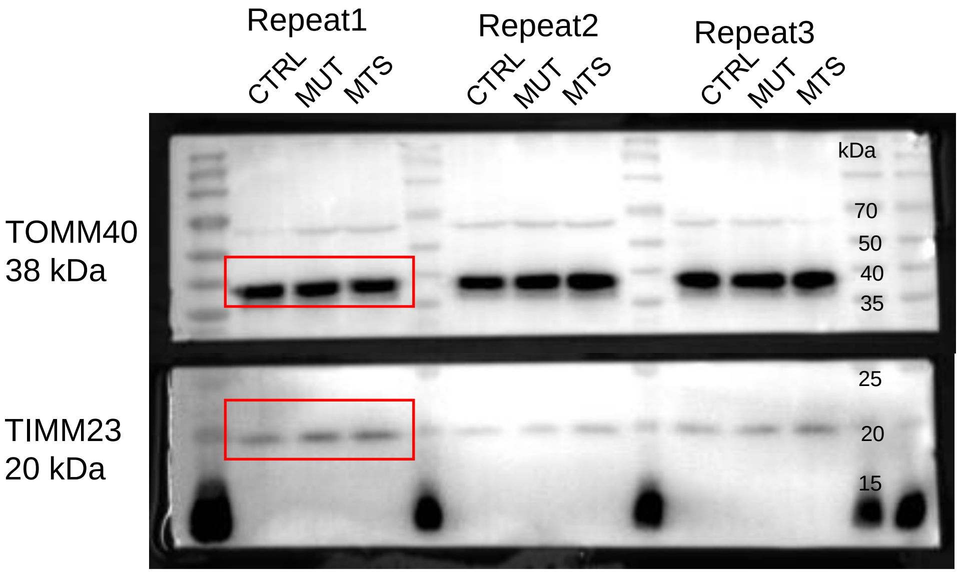

Supplementary Figure 3. Original western blotting images for Figure 3A

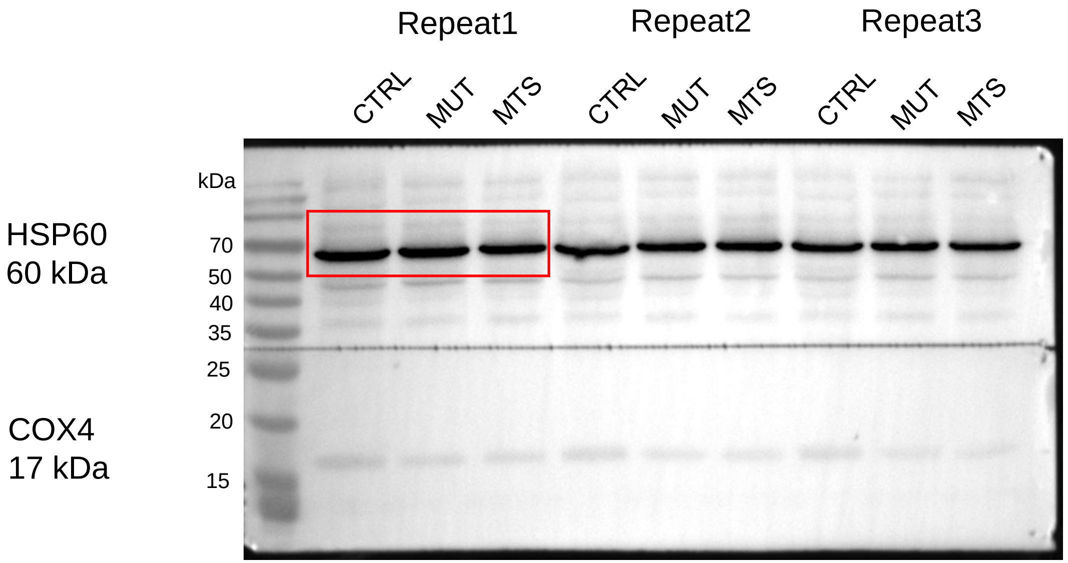

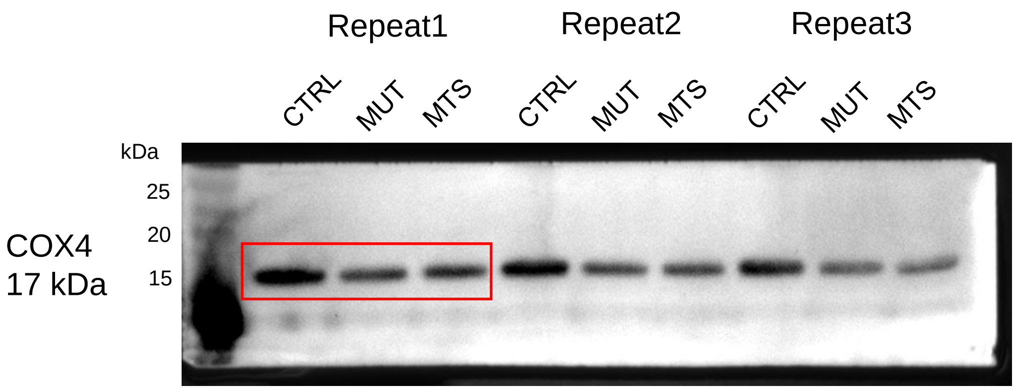

Supplementary Figure 4. Original western blotting images for Figure 3C

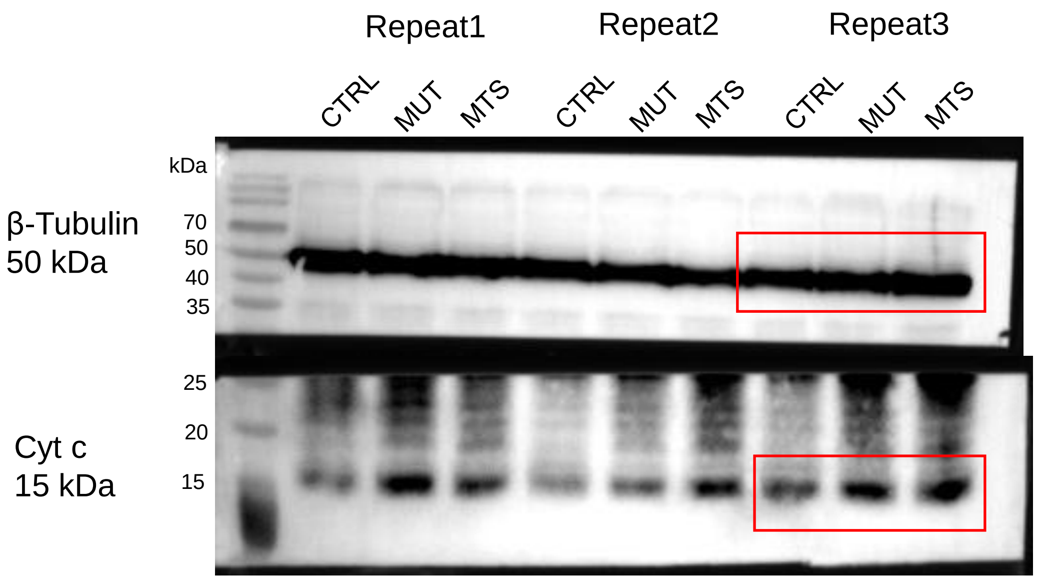

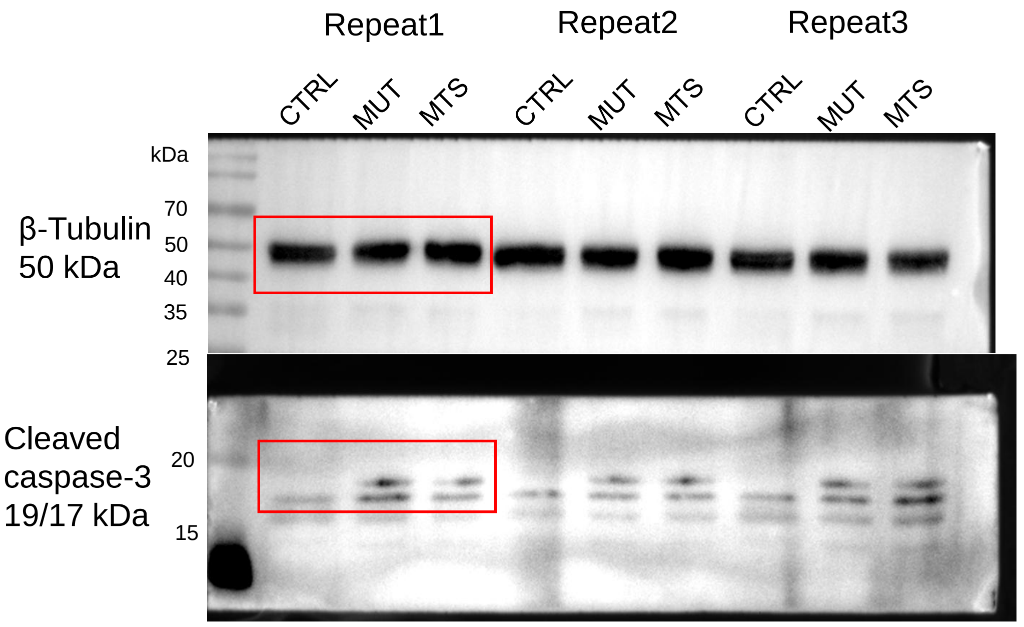

Supplementary Figure 5. Original western blotting images for Figure 3H

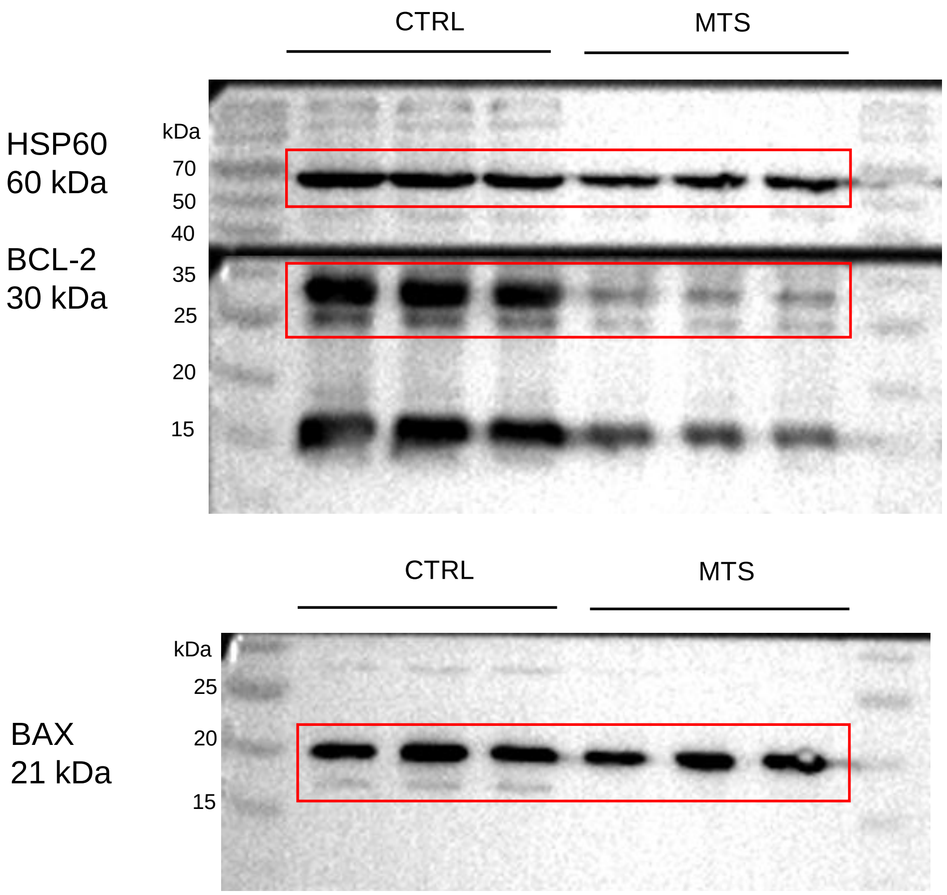

Supplementary Figure 6. Original western blotting images for Figure 4E

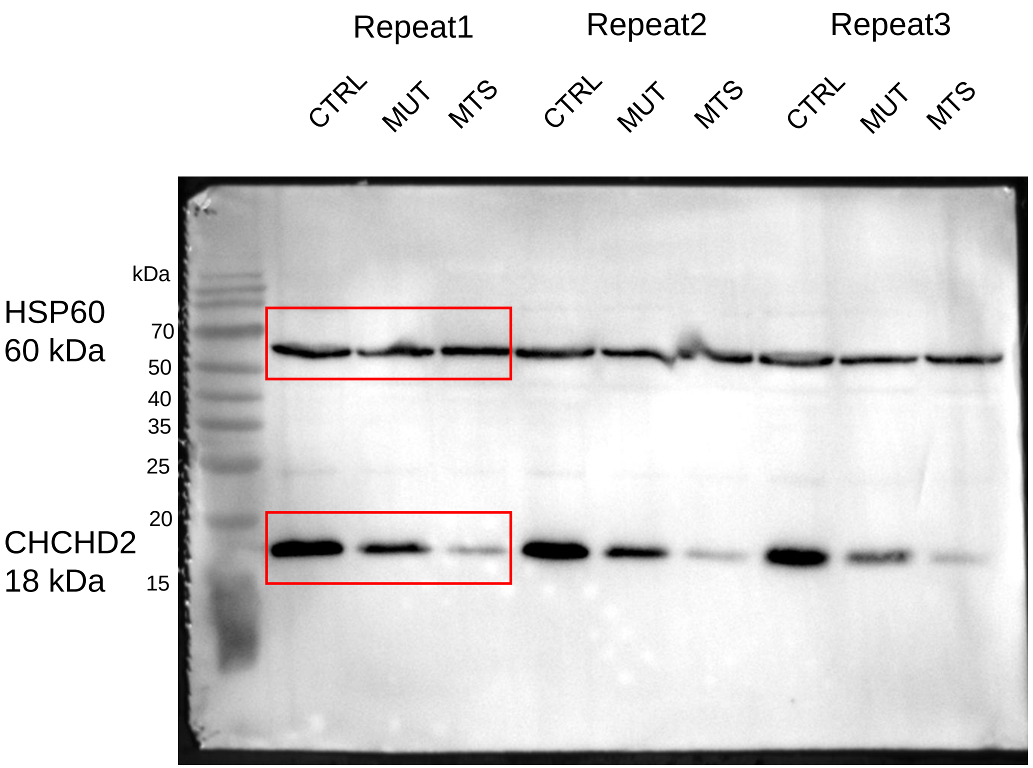

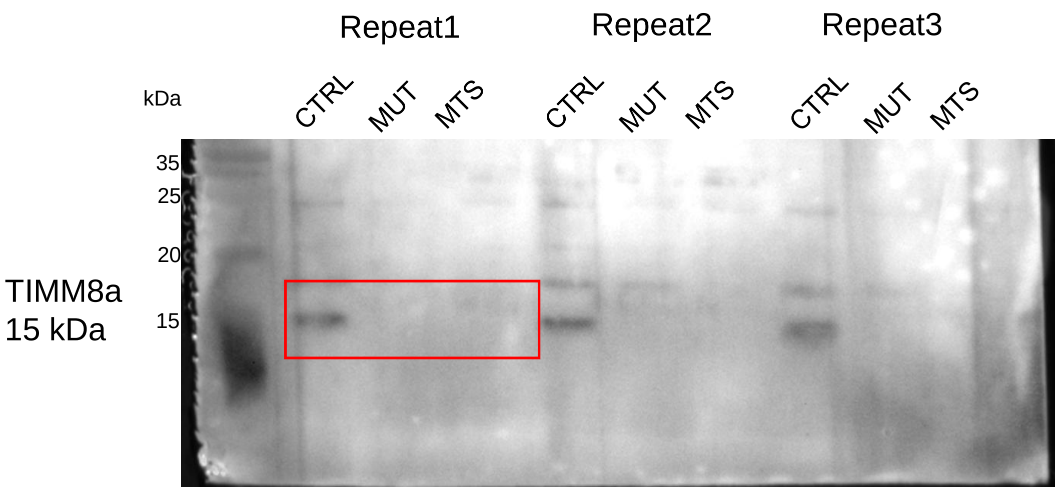

Supplementary Figure 7. Original western blotting images for Figure 4F

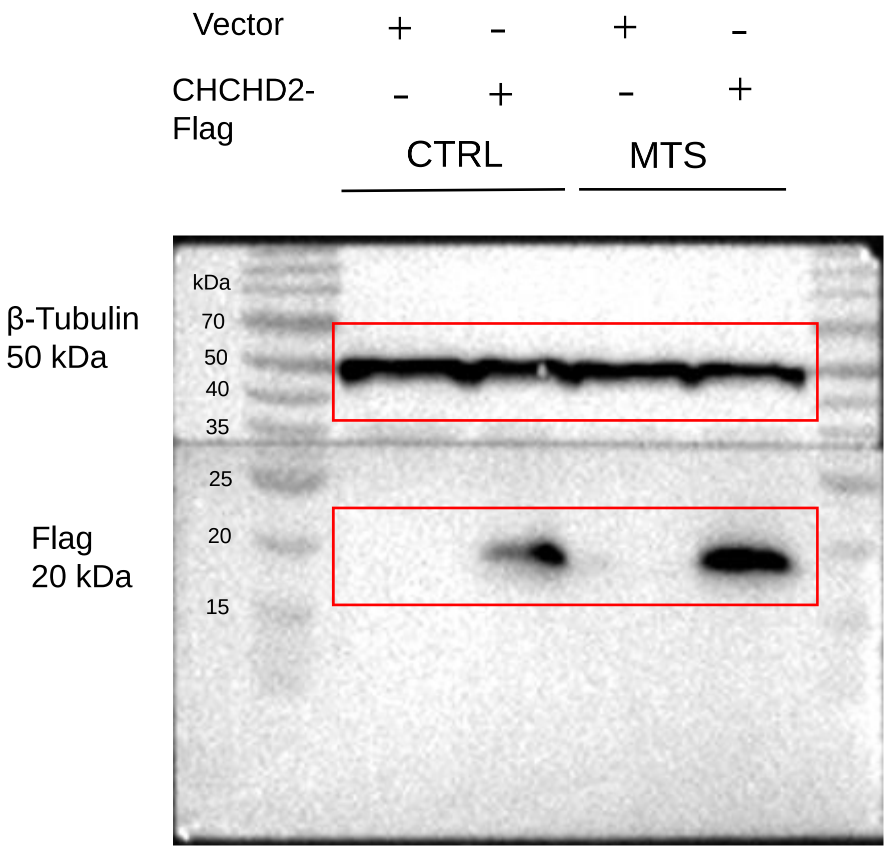

Supplementary Figure 8. Original western blotting images for Figure 5A

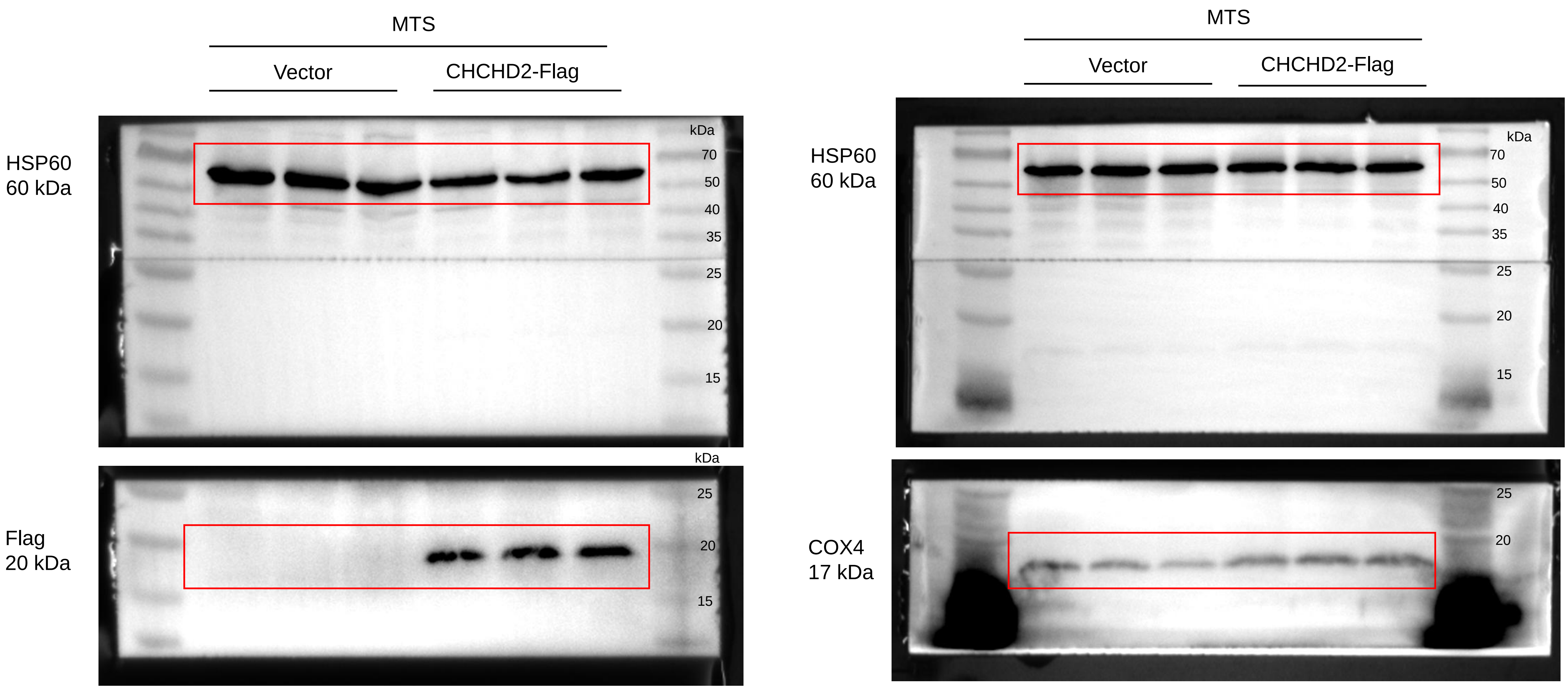

Supplementary Figure 9. Original western blotting images for Figure 5F

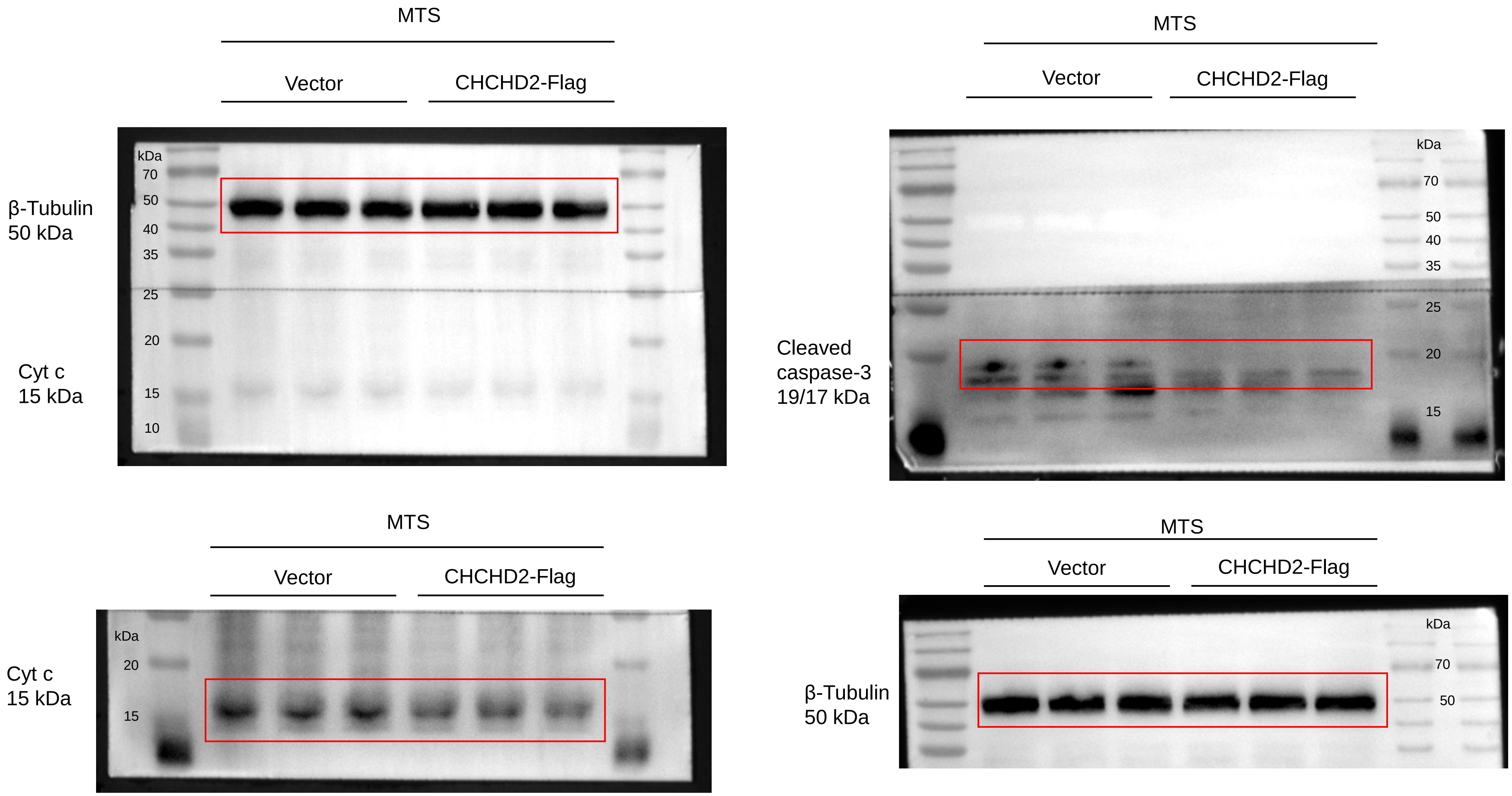

Supplementary Figure 10. Original western blotting images for Figure 5G
