# Supplementary material for: CHCHD2 rescues the mitochondrial dysfunction in iPSC-derived neurons from patient with Mohr-Tranebjaerg syndrome
Source: Cell Death Dis. 2025 Mar 12;16(1):173. doi: 10.1038/s41419-025-07472-9 (PMC11903874; doi:10.1038/s41419-025-07472-9)
Supplement: Supplementary file 6 — Legends for supplementary materials [file 41419_2025_7472_MOESM6_ESM.docx]

**Legends for supplementary materials**

**Table S1. Differentially expressed genes between the two groups related to Figure 4A.**

This file includes the detailed information of 2843 up-regulated and 1667 down-regulated genes between the two groups related to Figure 4A.

**Table S2**. **1836 Gene ontology biological process related to Figure 4B**

This file includes the detailed information of the significantly changed biological process enriched in Gene ontology analysis between the two groups related to Figure 4B.

**Table S3**. **111 KEGG pathways related to Figure 4C**

This file includes the detailed information of the significantly changed KEGG pathways between the two groups related to Figure 4C.

**Table S4**. **Differentially expressed genes related to apoptotic mitochondrial changes related to Figure 4D.**

This file includes the detailed information of differentially expressed genes related to apoptotic mitochondrial changes related to Figure 4D.

**Images of full and uncropped Western blots**

This file includes the original images of Western blot in the figures.
